# Efficient sensory coding of multidimensional stimuli

**DOI:** 10.1371/journal.pcbi.1008146

**Published:** 2020-09-24

**Authors:** Thomas E. Yerxa, Eric Kee, Michael R. DeWeese, Emily A. Cooper

**Affiliations:** 1 Department of Physics, University of California, Berkeley, Berkeley, California, United States of America; 2 Uber Advanced Technologies Group, Pittsburgh, Pennsylvania, United States of America; 3 Helen Wills Neuroscience Institute, University of California, Berkeley, Berkeley, California, United States of America; 4 Redwood Center for Theoretical Neuroscience, University of California, Berkeley, Berkeley, California, United States of America; 5 School of Optometry & Vision Science Program, University of California, Berkeley, Berkeley, California, United States of America; University of Pennsylvania, UNITED STATES

## Abstract

According to the efficient coding hypothesis, sensory systems are adapted to maximize their ability to encode information about the environment. Sensory neurons play a key role in encoding by selectively modulating their firing rate for a subset of all possible stimuli. This pattern of modulation is often summarized via a tuning curve. The optimally efficient distribution of tuning curves has been calculated in variety of ways for one-dimensional (1-D) stimuli. However, many sensory neurons encode multiple stimulus dimensions simultaneously. It remains unclear how applicable existing models of 1-D tuning curves are for neurons tuned across multiple dimensions. We describe a mathematical generalization that builds on prior work in 1-D to predict optimally efficient multidimensional tuning curves. Our results have implications for interpreting observed properties of neuronal populations. For example, our results suggest that not all tuning curve attributes (such as gain and bandwidth) are equally useful for evaluating the encoding efficiency of a population.

## Author summary

Our brains are tasked with processing a wide range of sensory inputs from the world around us. Natural sensory inputs are often complex and composed of multiple distinctive features (for example, an object may be characterized by its size, shape, color, and weight). Many neurons in the brain play a role in encoding multiple features, or dimensions, of sensory stimuli. Here, we employ the computational technique of population modeling to examine how groups of neurons in the brain can optimally encode multiple dimensions of sensory stimuli. This work provides predictions for theory-driven experiments that can leverage emerging high-throughput neural recording tools to characterize the properties of neuronal populations in response to complex natural stimuli.

## Introduction

A functioning nervous system is metabolically expensive, but offers a significant advantage in evolutionary fitness. This observation motivates the idea that natural selection has acted to optimize the nervous system subject to biological constraints. Indeed, optimality has proven to be a powerful concept for explaining the function of neural systems, circuits, and individual cells. But optimality arguments come with their own set of auxiliary questions. Optimal in what sense? Subject to what specific constraints?

A common framework for choosing these criteria is the *efficient coding hypothesis*. This hypothesis posits that sensory neurons maximize the information that they encode about the world while using as few resources (e.g., action potentials) as possible [1, 2]. For example, by defining a set of neurons as optimal if they may be used in linear combination to accurately represent an arbitrary image, and imposing the constraint that for any given input as little neural activity should be used as possible, we arrive at a sparse coding model [3]. Considering instead the constraint that each neuron should encode as little redundant information as possible gives rise to independent components analysis [4, 5].

**Fig 1:**
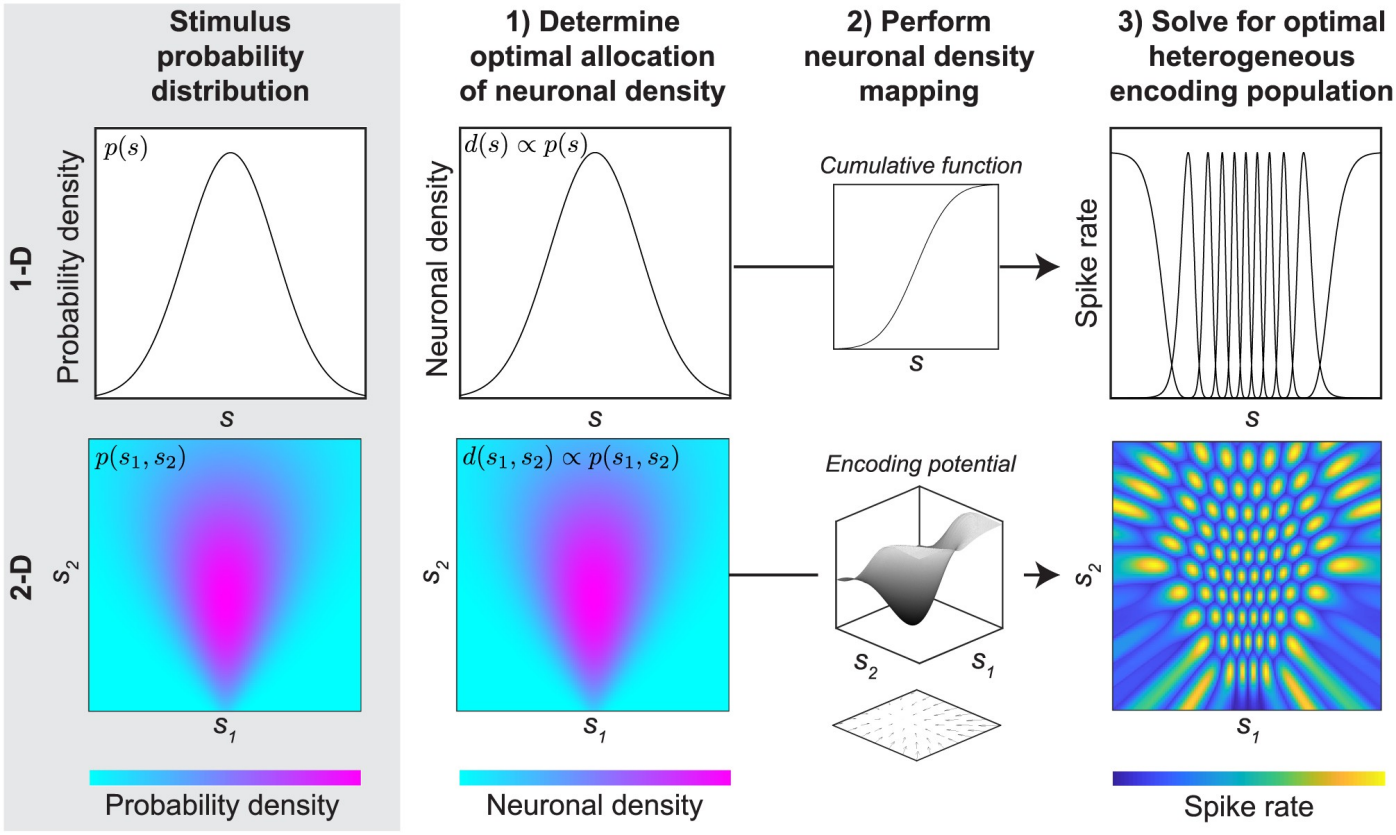
Overview of optimally efficient heterogeneous neuronal encoding populations in 1-D and 2-D. Top row: Based on prior work [8, 9], we derive a closed-form solution for an optimal heterogeneous neuronal population encoding a non-uniform 1-D stimulus probability distribution. This population has a neuronal density that is proportional to the probability of the stimulus. The neuronal density can be mapped to a specific population (here, represented by a set of Gaussian tuning curves) by using the cumulative of the probability distribution *p*(*s*) to warp the stimulus coordinates (*s*) over which each neuron’s tuning curve is defined. The resulting heterogeneous 1-D neuronal population is compressed in regions of higher probability and expanded in regions of lower probability. Bottom row: Neurons throughout the nervous system encode more than one stimulus dimension. Here, we extend the previous 1-D framework to examine optimally efficient neuronal populations in arbitrary dimensions. In closed form, we show that given the same assumptions used for 1-D populations, higher dimensional neuronal populations should also have density proportional to probability. However, in higher dimensions with statistical dependencies, density remapping cannot be achieved via the cumulative. Instead, we show that in both 1-D and higher dimensions, the optimal mapping is embodied by the gradient of a scalar potential function that reflects the displacement necessary to uniformly distribute neuronal density as a function of probability. This encoding potential is illustrated here as a surface, with its inverse gradient illustrated as a vector field below. The vector field is inverted to provide a more intuitive visual of how neuronal density gets condensed to areas of high probability. This gradient can be numerically optimized in 2-D for a given density function, which we exploit to derive optimal 2-D neuronal populations.

To understand how neuronal populations can operate efficiently during natural behavior, it is often advantageous to consider that natural sensory signals have robust statistical regularities (See [6] and [7] for review). Thus, neurons are generally tasked with encoding a small subset of all possible stimuli. Said another way, the probability distribution of stimuli experienced in the natural environment is highly non-uniform. Given a particular non-uniform probability distribution of a 1-D stimulus, a body of recent work considers how to optimize the efficiency of neuronal populations in which each neuron is characterized by a tuning curve that defines its mean response as a function of the stimulus value [8–14]. This simple population model yields several appealing solutions for optimal encoding populations with testable predictions. For example, a recent formulation predicts that neurons tuned to more frequently occurring stimulus values should be more densely packed and tightly tuned, but should not necessarily have higher or lower response gain (Fig 1, upper row) [8, 9]. These predictions with respect to density and tuning shape can successfully explain empirical measurements in a variety of sensory neuron populations [15–17].

While 1-D population models have yielded a range of insights and predictions for neural efficiency, it is important to note that sensory neurons throughout the brain are selective to more than one stimulus dimension. Canonical examples include neurons in mammalian primary visual and auditory cortices that can jointly encode spatial frequency/orientation and pitch/timbre, respectively [18, 19], as well as medial temporal neurons that encode multiple variables relevant to 3-D spatial position or pose [20–23]. In higher-level sensory cortices, larger dimensionality in the encoding regimes may be common (e.g., [24, 25]). To date, the pay off of the efficient coding framework for understanding high-dimensional stimulus encoding has been more modest because fewer predictions for efficient multidimensional tuning curves are formalized [13, 26]. As a consequence, it is more challenging to predict and evaluate efficiency in higher dimensions. With high-throughput experimental paradigms and large data sets becoming more common in neuroscience, the ability to model optimal encoding of higher dimensional stimuli is increasingly relevant.

In the current work, we make advances towards filling this gap by extending existing frameworks for modeling efficient 1-D population codes to higher dimensions (summarized in Fig 1, bottom row). We first review the formulation of an encoding model that employs a constrained heterogeneous population of neurons to optimally (in the information preserving sense) encode a 1-D stimulus using a finite and fixed number of spikes [8, 9]. As our first contribution, we generalize this formulation by re-parameterizing the heterogeneous population in terms of a more generic transformation, based on recent work describing transformations between probability distributions [27]. This generalization replicates prior work in 1-D and enables a natural extension to solve for the parameters of optimal encoding populations in higher dimensions. We show that this extension produces similarly elegant predictions for the relationship between stimulus probability and neuronal density in populations encoding an arbitrary number of stimulus dimensions. However, predicting the actual shapes of multidimensional tuning curves when there are statistical dependencies between stimulus dimensions remains more complicated. Focusing on 2-D stimuli, we show that it is nonetheless possible to utilize a numerical method (developed in [27]) to predict tuning curves of efficient encoding populations across the stimulus space. Using this approach, we examine the predicted tuning properties for neuronal encoding populations optimized for 2-D stimuli. These results highlight both the utility and limitations of simple encoding models for evaluating the efficiency of neuronal populations that encode multiple stimulus dimensions.

## Methods

### Encoding objective

One natural question in the context of neural coding is: how much information about a stimulus is contained in the responses of a population of sensory neurons? The average amount of information learned about some random variable (say, the value of a stimulus) by observing another random variable (say, the firing rate of a sensory neuron) is given by the mutual information (M[x,y]) [28]:

$$\begin{array}{c} M\left[x,y\right]=\int \int p\left(x,y\right)\text{log}(\frac{p(x,y)}{p(x)p(y)})dxdy. \end{array}$$ (1)

Here, *x* and *y* denote any two random variables, *p*(⋅) denotes the probability distribution of a variable, and *p*(*x*, *y*) denotes their joint probability. Though mutual information appears to be a strong candidate for an objective function to maximize for efficiency, it is difficult to optimize because doing so requires integration over all variables. To avoid this issue, researchers often instead optimize approximations of the mutual information that make use of a quantity called the Fisher information (F) [9, 26, 28–30]. The Fisher information provides another way to quantify the amount of information an observable random variable (*x*) carries about a parameter (*y*). It can be computed from the conditional probability distribution of *x* given *y* as

$$\begin{array}{c} F\left(y\right)=E_{y}\left[-\frac{\partial^{2}\text{log}\left(p\left(x|y\right)\right)}{\partial y^{2}}\right], \end{array}$$ (2)

where *E*_*y*_[⋅] denotes taking the expectation of the bracketed property over the parameter *y*. When the curvature of the conditional distribution, *p*(*x*|*y*), is high, small changes in the parameter cause large changes in the probability. Thus, observations in this region are more informative.

The Fisher information limits the variance with which a parameter may be estimated (through a relationship known as the Cramer-Rao bound). This fact has been used to derive an approximation of mutual information [11, 12, 31]:

$$\begin{array}{c} M\left[x,y\right]\geq H\left(y\right)+\int p\left(y\right)\frac{1}{2}\text{log}(\frac{F(y)}{2\pi e})dy. \end{array}$$ (3)

Here H(y) is the entropy of the probability distribution for the parameter *y*, and is a constant. For an unbiased estimator, this approximation approaches equality in the limit of low noise, which occurs in the context of neural coding when there are either a large number of neurons or a large number of action potentials fired. A recent and detailed exploration of Fisher information and mutual information can be found in [12]. Following previous work, we will thus use the Fisher information in the neuronal population response to the sensory stimulus to determine the quantity to be maximized in our neuronal population model [8–11, 13, 14, 31–33].

### Neuronal population model

The encoding characteristics of sensory neurons are often described via a tuning curve, which is a function that defines how an idealized neuron responds to different values of a particular stimulus. In this work, we will consider neuronal responses in the form of rate codes, where the tuning curve of a neuron defines the mean firing rate as a function of the presented stimulus value. There is an inherent trade off between the range of stimulus values that an individual neuron can encode and how much information it can convey. A very broad tuning curve would be able to respond to a large number of stimulus values, but because the magnitude of the response changes slowly it will be more difficult to discriminate between different encoded values. Conversely a very narrow tuning curve might be able to encode a single value with very high certainty, but would leave a large set of values unaccounted for. In practice, sensory systems often use a large population of neurons (each with their own, different tuning curve) to encode the same sensory variable. This strategy is known as population coding.

We will consider a neuronal population in which each neuron’s firing rate is determined by an independent Poisson process. In describing this population, we adopt notation from [9], as our work builds on theirs. A (homogeneous) Poisson process is a model for a series of independent events (in this case, action potentials) in which events are equally likely to occur at any time independent of the number or timing of previous events. The distribution that describes the probability of observing *r* events within a given time interval for a Poisson process is defined by a single parameter, λ, which represents the expected (mean) number of events in the interval. The probability distribution for observing *r* events for a Poisson process with mean λ is:

$$\begin{array}{c} p\left(r\right)=\frac{\lambda^{r}e^{-\lambda }}{r!}. \end{array}$$ (4)

In the context of neural coding, the parameter λ that determines the mean firing rate *r* of a neuron in response to a stimulus is defined by that neuron’s tuning curve *h*(⋅). Since neurons can be tuned over multiple stimulus dimensions, we can denote the stimulus attributes that modulate the firing rate with the vector ***s*** of any dimensionality. However, we will initially be focusing on the case of 1-D stimuli, so we will denote the 1-D stimulus as *s* and the tuning curve as *h*(*s*). Thus, the probability of a sensory neuron firing with rate *r* conditioned on the presentation of the stimulus *s* is:

$$\begin{array}{c} p\left(r|s\right)=\frac{h{\left(s\right)}^{r}e^{-h(s)}}{r!}. \end{array}$$ (5)

We will consider a population of *N* neurons, each with its own tuning curve, which respond to the presented stimulus (conditionally) independently of all of the other neurons in the population. Thus, the probability of observing a population response ***r*** upon the presentation of stimulus *s* is:

$$\begin{array}{c} p\left(r|s\right)=\prod_{n}^{N}\frac{h_{n}{\left(s\right)}^{r_{n}}e^{-h_{n}\left(s\right)}}{r_{n}!}. \end{array}$$ (6)

Here *n* indexes the population of neurons, *h*_*n*_ is the tuning curve of the *n*^*th*^ neuron, and ***r*** is an N-dimensional continuous vector whose elements (*r*_*n*_) denote the firing rate of the *n*^*th*^ neuron in response to some stimulus. Because each neuron is assumed to act conditionally independently of any other neuron in the population, the joint probability of the responses is modeled as the product of the individual responses.

The primary goal of this work will be to find how a population of sensory neurons should respond to the presentation of a stimulus in order to maximize the mutual information between the stimulus and response. To do so we will optimize an approximation of M[r,s]. As such, we will use the Fisher information for the independent Poisson population encoding model. As described in [33], this quantity is given by:

$$\begin{array}{c} F\left(s\right)=\sum_{n}^{N}\frac{h_{n}^{{}^{\prime }2}\left(s\right)}{h_{n}\left(s\right)}, \end{array}$$ (7)

where $h_{n}^{\prime }\left(s\right)$ denotes the first derivative of the tuning curve.

### Parameterization of 1-D heterogeneous neuronal populations

**Fig 2:**
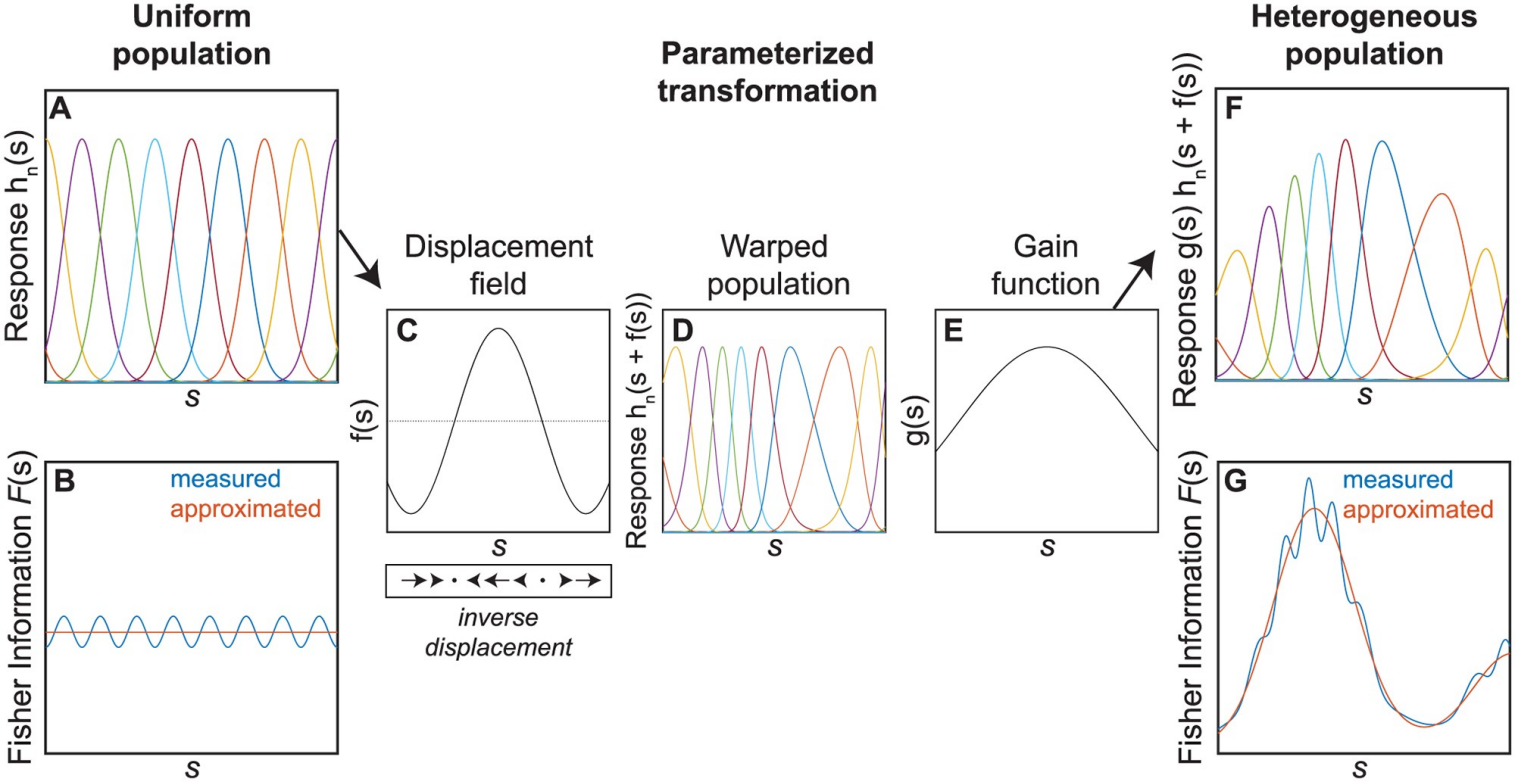
Parameterizing hetereogeneous neuronal populations. A) A uniform population of neurons approximately tiles the stimulus space (*s*) with identical, equally spaced Gaussian tuning curves. B) The Fisher information of this population is roughly uniform (blue line), matching the approximation in Eq (16) (red line). C) A displacement field that is smooth and slowly varying relative to the tuning curves. These displacement values apply to the stimulus space, arrows below illustrate the direction and magnitude of shifts in the resulting tuning curves defined over *s* (which corresponds to the inverse of the displacement field). D) After the displacement field is applied, the neuronal population now has heterogeneous tuning curves. Displacements that stretch the stimulus space result in denser, narrower tunings. Displacements that compress the stimulus space result in sparser, wider tunings. E) A gain function that is smooth relative to the tuning curves can also allow neurons to have different response magnitudes. F) Following the application of both the displacement field and the gain function, we have a transformed heterogeneous population with variable tuning curves. G) The Fisher information in the hetereogenous population is no longer uniform, as illustrated by the measured (blue) and approximated (red) lines. S1 Fig illustrates the consequences when the displacement field and gain function are not smooth and slowly varying with respect to the tuning curves.

Our parameterization of hetereogeneous populations follows on the formulation introduced in [8, 9]. As a starting point (Fig 2), we will define a population of 1-D tuning curves, where each curve has an identical shape determined by the function *h*(*s*), and individual tuning curves (*h*_*n*_(*s*)) fall at evenly spaced intervals so that *h*_*n*_(*s*) = *h*(*s* − *s*_*n*_). Here, *s*_*n*_ denotes evenly spaced values that determine the preferred stimulus for each neuron (Fig 2A). Additionally, we assume that the population of neurons approximately tiles the stimulus space (tuning curves are thus not separated by large gaps, nor do they take the form of monotonically increasing functions) [8, 9, 34, 35]. Specifically,

$$\begin{array}{c} \sum_{n}^{N}h_{n}\left(s\right)\approx k. \end{array}$$ (8)

This means that the number of spikes emitted by the population is an approximately constant (*k*) function of the presented stimulus. The constant spike rate means that the Fisher information for this uniform population (which we denote $F_{\;u}\left(s\right)$) is also approximately constant (Eq (7), Fig 2B). Therefore, this population is non-optimal for encoding stimuli with non-uniform probability distributions.

To determine an optimal encoding population for a non-uniformly distributed stimulus, we will define two functions that warp and scale this uniform population, and thus reallocate Fisher information to different regions of the stimulus space. The choice of parameters and the constraints on their smoothness build on the formulation introduced in [8, 9]. However, it is important to note that alternative parameterizations can produce quite different predictions [12, 30], which we will consider in the Discussion.

Specifically, we will parameterize the population with two smooth and continuous functions. The first is a displacement field, *f*(*s*), which will warp the tuning curves to change their local density (Fig 2C and 2D). The second is a gain function, *g*(*s*), which will control the maximum response of neurons to a given stimulus (by multiplicatively scaling the size of the response) (Fig 2E and 2F). Each of these functions will also be constrained to be slowly changing relative to the individual tuning curves. This property of each parameter will force the warped and scaled tuning curves to (approximately) retain their functional form and tile the space (S1 Fig illustrates the consequences of violating this constraint).

The notable difference between this formulation and that of previous work [8, 9] is that we use a displacement field to change neuronal density. The displacement field can be thought of as transforming the original stimulus space (which is simply the value of some stimulus variable *s*) to a new domain as follows:

$$\begin{array}{c} \hat{s}=s+f\left(s\right). \end{array}$$ (9)

Following the nomenclature suggested by [30], we will refer to the original space as the stimulus space (which is the space defined by the stimulus variable *s*), and the transformed space as the sensory space ($\hat{s}$, which is the space defined by some transformation as denoted in Eq (9)). In contrast to this approach, previous work changed neuronal density by applying the cumulative of a function that directly described the desired density of tuning curves. Shifting the population based on the cumulative of the desired density function has the elegant property of producing the desired density directly [8, 9, 30, 36]. However, the reliance on a cumulative function is not appropriate when considering higher dimensions with statistical dependencies, because the multivariate cumulative treats each dimension separately. The more general displacement field is still related to the density function, and in 1-D there is still a one-to-one relationship between displacement and density.

What is the relationship between the transformation in Eq (9) and density in 1-D? The first derivative of a 1-D transformation directly embodies the change in density associated with that transformation (indeed, this is why the cumulative of the desired density function was used in prior work). Thus, if the density with which the tuning curves tile the space is defined by *d*(*s*), then in 1-D we simply take the first derivative of Eq (9) to obtain the density after transformation:

$$\begin{array}{c} d\left(s\right)=1+f^{\prime }\left(s\right). \end{array}$$ (10)

Intuitively, this relationship arises because *f*(*s*) defines how much each point on each tuning curve is shifted in a given direction, and successive points being shifted in the same direction by an increasing amount will become less densely packed. Similarly, successive points being shifted in the same direction by a decreasing amount will become more densely packed.

### Fisher information in the 1-D heterogeneous neuronal populations

The displacement field and gain function each have a distinct effect on the Fisher information of the population. The effect of the displacement field on the Fisher information is:

$$\begin{array}{c} F_{\;f}\left(s\right)=F_{\;u}\left(\hat{s}\right){\left(\frac{d\hat{s}}{ds}\right)}^{2}. \end{array}$$ (11)

Here, $F_{\;f}\left(s\right)$ denotes the Fisher information after the stimulus space has been ‘warped’ into the sensory space. Because $F_{\;u}\left(\cdot \right)$, the Fisher information of the unwarped uniform population, is approximately equal to a constant, we can rewrite this equation with the constant denoted as *U*:

$$\begin{array}{c} F_{\;f}\left(s\right)\approx U{\left(1+f^{\prime }\left(s\right)\right)}^{2}. \end{array}$$ (12)

Here, we have also rewritten the first derivative of $\hat{s}$ with respect to *s* following Eq (9), which we already noted is equal to the neuronal density (Eq (10)).

To consider the effect of the gain parameter on the Fisher information, we multiply each of the tuning curves in Eq (7) by a function *g*(*s*), giving:

$$\begin{array}{c} F_{\;g}\left(s\right)=\sum_{n}^{N}\frac{{\left[{\left[g\left(s\right)h_{n}\left(s\right)\right]}^{\prime }\right]}^{2}}{g\left(s\right)h_{n}\left(s\right)}. \end{array}$$ (13)

Then using the product rule we see,

$$\begin{array}{c} F_{\;g}\left(s\right)=\sum_{n}^{N}\frac{{\left(g\left(s\right)h_{n}^{\prime }\left(s\right)+g^{\prime }\left(s\right)h_{n}\left(s\right)\right)}^{2}}{g\left(s\right)h_{n}\left(s\right)}. \end{array}$$ (14)

Assuming that the gain function *g*(*s*) changes slowly compared to the tuning curves, so *g*(*s*)*h*′(*s*) ≫ *g*′(*s*)*h*(*s*), we have:

$$\begin{array}{c} F_{\;g}\left(s\right)\approx \sum_{n}^{N}\frac{{\left(g\left(s\right)h_{n}^{\prime }\left(s\right)\right)}^{2}}{g\left(s\right)h_{n}\left(s\right)}=g\left(s\right)U. \end{array}$$ (15)

So, the Fisher information of the warped and scaled population $F_{f,g}\left(s\right)$ is:

$$\begin{array}{c} F_{f,g}\left(s\right)\approx g\left(s\right){\left(1+f^{\prime }\left(s\right)\right)}^{2}U. \end{array}$$ (16)

These approximations apply for the Fisher information of the population, rather than for any individual tuning curve. The validity of the assumptions we made to arrive at this expression for the Fisher information can be visualized by comparing the result of Eq (16) to the empirically measured Fisher information in example neuronal populations. We can see in Fig 2B and 2G that the uniform population Fisher information is approximately constant and that the example warped and scaled population Fisher information is well described by Eq (16).

## Results

### Optimally efficient 1-D heterogeneous neuronal populations

Using the described coding objective and neuronal population model, we will first determine the parameters of a neuronal population optimized to encode a 1-D stimulus. The constants in Eq (3) can be ignored for the purpose of optimization. So the final form of the objective function we will use is:

$$\begin{array}{c} \int p\left(s\right)\text{log}[F_{f,g}\left(s\right)]ds. \end{array}$$ (17)

In accordance with previous work on this problem [8, 9] we adopt two constraints:

∫p(s)g(s)ds=R (18)

$$\int (1+f^{\prime }\left(s\right))ds=N.$$ (19)

These are global resource constraints. The first is approximately equal to the expected firing rate of the population as a whole and so can be interpreted as requiring that the total number of spikes fired by the population in a given time interval will be finite and constant. Integrating the density of tuning curves yields the total number of neurons in the population, thus the second constraint expresses the fact that the encoding scheme must only use a finite number of neurons.

We are dealing with an optimization problem with integral constraints so we turn to methods from the calculus of variations. To solve such a problem, as in [8, 9], we design a function called a Lagrangian,

$$\begin{array}{c} \begin{array}{cc} & L(f^{\prime }\left(s\right),g\left(s\right),s,\lambda_{1},\lambda_{2})=\\ & \int p\left(s\right)\text{log}\left[g{\left(1+f^{\prime }\left(s\right)\right)}^{2}U\right]ds+\lambda_{1}(\int (1+f^{\prime }\left(s\right))ds-N)+\lambda_{2}(\int p(s)g(s)ds-R). \end{array} \end{array}$$ (20)

To optimize, we solve the system of equations obtained by setting the gradient of the Lagrangian equal to zero (note that in this case taking the partial derivative with respect to the Lagrange multipliers λ_1_ and λ_2_ simply reproduces the constraint equations):

$$\begin{array}{cc} & \frac{\partial L}{\partial f^{\prime }\left(s\right)}=\frac{2p(s)}{1+f^{\prime }\left(s\right)}-\lambda_{1}=0 \end{array}$$ (21)

$$\begin{array}{cc} & \frac{\partial L}{\partial g(s)}=\frac{p(s)}{g(s)}+\lambda_{2}p\left(s\right)=0. \end{array}$$ (22)

Solving for 1+ *f*′(*s*) in the first equation and *g*(*s*) in the second gives:

$$1+f^{\prime }\left(s\right)=\frac{2p(s)}{\lambda_{1}}$$ (23)

$$g(s)=\frac{1}{\lambda_{2}}.$$ (24)

Because 1+ *f*′(*s*) must integrate to *N*, *p*(*s*) integrates to 1, and *p*(*s*)*g*(*s*) must integrate to *R*, it is simple to determine the value of the λ’s and write the solutions:

$$1+f^{\prime }\left(s\right)=Np(s)$$ (25)

g(s)=R. (26)

Recall that 1+ *f*′(*s*) is equivalent to the density function from prior work, which we denote with *d*(*s*) (Eq (10)) [8, 9]. Thus, substituting this identity in to Eq (25), we reach the same conclusion as prior work that

$$\begin{array}{c} d(s)=Np(s). \end{array}$$ (27)

That is, the density of tuning curves in the optimal encoding population is proportional to the probability distribution of the stimulus and the gain is constant (see Fig 1 upper row).

Because of the resulting proportionality between probability and density, and the fact that the gain function should be held constant, the optimal transformation results in a population where each neuron is equally likely to fire. In regions of high stimulus probability, there are a large number of tuning curves that are relatively narrow or selective. In regions of low probability, the tuning curves are sparser, but more broad (so they fire strongly in response to a larger range of stimuli). Said another way, the transformation to the optimal population maps the stimulus distribution to a new sensory space in which the distribution and the Fisher information are all uniform. This observation links the problem of optimal coding to the problem of finding a *mapping function* between an arbitrary probability distribution and the uniform distribution.

What is the form of the mapping function that accomplishes this goal? Prior work defined this mapping function *a priori* to be the cumulative of a function parameterizing the density of the tuning curves [8, 9, 30]. Following Eq (27), the optimal mapping function is then simply a scaled version of the cumulative of *p*(*s*). However, by using the cumulative density, this formulation is limited to situations in which the transformation *can* be described by a cumulative. This limitation becomes problematic for the case of multidimensional stimuli because the cumulative treats all dimensions as if they are separable. In our new formulation, we have replaced the cumulative density with the more general transformation embodied by the variable $\hat{s}$ (that is, *s*+ *f*(*s*)). This generalization produces the same solution in 1-D, as described above. In addition, in the following section we will see that this formulation can describe the appropriate allocation of neuronal density for non-separable distributions in arbitrary dimensions in closed form.

### Extending the 1-D optimization to arbitrary dimensions

For a neuronal population encoding *k* stimulus dimensions, the Fisher information becomes a *k*x*k* matrix with the entry in row i and column j defined by:

$$\begin{array}{c} {\left[F(s)\right]}_{ij}=E{\left[-\frac{\partial^{2}\text{log}\;p\left(r|s\right)}{\partial s_{i}\partial s_{j}}\right]}_{s}, \end{array}$$ (28)

where *s*_*i*_ and *s*_*j*_ are two dimensions in the stimulus space. Note that the multidimensional stimulus is now defined by a *k*-dimensional vector ***s***. The *k*-dimensional tuning function of the *n*th neuron in the population will thus be written as *h*_*n*_(***s***). For the Poisson population, Eq (28) reduces to:

$$\begin{array}{c} {\left[F(s)\right]}_{ij}=\sum_{n}\left(\frac{\partial h_{n}\left(s\right)}{\partial s_{i}})(\frac{\partial h_{n}\left(s\right)}{\partial s_{j}})(\frac{1}{h_{n}\left(s\right)}\right). \end{array}$$ (29)

The objective function will now depend on the determinant of the Fisher information matrix, rather than the scalar value of the Fisher information. This is the case because in 1-D Fisher information bounds a variance, while in multiple dimensions the determinant of the Fisher information matrix bounds the determinant of a covariance matrix [11]. Thus, similar to the 1-D population (Eq (17)), the *k*-dimensional population will seek to optimize

$$\begin{array}{c} \int p\left(s\right)\text{log}(|F_{f,g}\left(s\right)|)ds, \end{array}$$ (30)

where |⋅| denotes the matrix determinant.

The expression for how the Fisher information changes under the multidimensional reparameterization must be adjusted from 1-D as well. Starting with the displacement transformation from Eq (9), which is now $\hat{s}=s+f\left(s\right)$, the effect of the warping into the sensory space is:

$$\begin{array}{c} F_{\;f}\left(s\right)=J_{\hat{s}}^{T}F_{\;u}\left(\hat{s}\right)J_{\hat{s}}, \end{array}$$ (31)

where $J_{\hat{s}}$ is the Jacobian matrix of the transformation from ***s*** to $\hat{s}$. For square matrices, |***AB***| = |***A***||***B***| and |***A***^*T*^| = |***A***|, so we can write

$$\begin{array}{c} |F_{\;f}\left(s\right)|=|F_{\;u}\left(\hat{s}\right)\left|\right|J_{\hat{s}}{|}^{2}. \end{array}$$ (32)

In 1-D we made the assumption that the sum of the Fisher information as a function of the stimulus variable was approximately constant, and the corresponding assumption in multiple dimensions is that the determinant of $F_{\;u}\left(\cdot \right)$ is approximately constant. We will again call this constant *U*.

The effect of the gain function on the Fisher information will also be different in higher dimensions. The gain function will multiply each element of the matrix, so because |*a**A***| = *a*^*k*^|***A***| where *k* is the dimension of the matrix, we have:

$$\begin{array}{c} |F_{\;g}{\left(s\right)|=g\left(s\right)}^{k}U. \end{array}$$ (33)

Finally, the determinant of the fully parameterized Fisher information matrix can be written as:

$$\begin{array}{c} |F_{f,g}{\left(s\right)|=g\left(s\right)}^{k}{\left|J_{\hat{s}}\right|}^{2}U. \end{array}$$ (34)

After making the replacement for the density of the warped tuning curves $d\left(s\right)=|J_{\hat{s}}|$ (note that the determinant of the Jacobian of a transformation *defines* the relative density between the two spaces), we can write the Lagrangian for the multidimensional case (the optimization constraints are essentially unchanged from the one dimensional case):

$$\begin{array}{c} \begin{array}{cc} & L(d(s),g(s),s)=\\ & \int p\left(s\right)\text{log}\left[g{\left(s\right)}^{k}{d(s)}^{2}U\right]ds+\lambda_{1}(\int d(s)ds-N)+\lambda_{2}(\int p(s)g(s)ds-R). \end{array} \end{array}$$ (35)

Setting the gradient equal to zero yields the system of equations:

$$\begin{array}{cc} & \frac{\partial L}{\partial d(s)}=\frac{2p(s)}{d(s)}-\lambda_{1}=0 \end{array}$$ (36)

$$\begin{array}{cc} & \frac{\partial L}{\partial g(s)}=\frac{p(s)}{g(s)}+\lambda_{2}p\left(s\right)=0, \end{array}$$ (37)

so we conclude that *d*(***s***) ∝ *p*(***s***) and *g*(***s***) ∝ a constant. This gives us a result in higher dimensions that mirrors the 1-D case:

d(s)=Np(s) (38)

g(s)=R. (39)

In summary, rather than parameterize a heterogenous population of tuning curves by the cumulative of the density with which they tile the stimulus space, we chose instead to use a displacement field that warps an initially uniform lattice. This choice allowed us to avoid any reference to a cumulative density function, which is beneficial because the utility of the cumulative is restricted to 1-D. However, because the objective function turns out to depend on the Jacobian of the transformation between the uniform tiling and the warped tiling, the density still appears in the *k*-dimensional optimization problem in a natural way. This allowed us to arrive at a solution for the optimal density with which a population of heterogeneous tuning curves should tile a multidimensional stimulus space given the probability distribution of the stimuli, and a solution for the gain function that optimally modulates the firing rate of each neuron in said population. Additionally, the result is still intuitively sensible: it says that the tiling should be more dense in regions where the stimulus is more likely, but that the response gain of tuning curves should be uniform. Taken together these statements imply that in an optimal population each neuron is equally likely to fire a spike in natural conditions.

Importantly, at this point we have not shown that a displacement field exists that can describe an optimal transformation between the stimulus space ***s*** and the sensory space $\hat{s}$. We have rather shown that if or when this displacement field does exist, the optimal density of a population of tuning curves is proportional to the density of the multidimensional stimulus. Note also that the precise arrangement of a *k*-dimensional population is not uniquely determined by the density because it only describes the *determinant* of the Jacobian, which cannot be integrated to recover the mapping. This is in contrast to 1-D, where the Jacobian is simply the 1-D derivative making it possible to directly integrate the density to recover the mapping. Thus for multidimensional tuning curves, in order to show that the optimal neuronal population density is proportional to the density of the stimulus, a field ***f***(***s***) that maps between stimulus and sensory space must be found such that its Jacobian determinant is properly defined. In the next section, we show that, although there is no longer a one-to-one correspondence between the density and the mapping function in higher dimensions, the mapping function is nonetheless well constrained and can be approximated with numerical methods.

### Determining optimally efficient heterogeneous neuronal populations in higher dimensions

A description of the multidimensional displacement field ***f***(***s***) that embodies the mapping function can be derived by considering the properties of the stimulus and sensory spaces. Following on the derivation in [27], we will show that some basic assumptions about these spaces can be used to constrain the possible transformations between them. First, we note that both the stimulus probability distribution in stimulus space *p*(***s***) and the uniform probability distribution in sensory space $u(\hat{s})$ must integrate to one. Thus, we have:

$$\begin{array}{c} \int p(s)ds\;=\;\int u\left(\hat{s}\right)d\hat{s}, \end{array}$$ (40)

where multiple integration over the dimensions of ***s*** and $\hat{s}$ is implied.

To examine the relationship between the two spaces, we can eliminate the integrals by differentiating both sides of this equation with respect to ***s***. First, because $\hat{s}$ is defined by a transformation of ***s***, we can let $\hat{c}\left(s\right)$ be the transformation function for sensory coordinates: $\hat{s}=\hat{c}\left(s\right)=s+f\left(s\right)$. The second integral can now be rewritten using the change of variables theorem yielding:

$$\begin{array}{c} \int p(s)ds\;=\;\int u\left(\hat{c}\left(s\right)\right)\left|J\left(\hat{c}\left(s\right)\right)\right|ds. \end{array}$$ (41)

Here J(⋅) denotes the Jacobian operator, and $J(\hat{c}\left(s\right))$ gives the Jacobian matrix $J_{\hat{s}}$ from Eq (31). Taking the derivative of each side with respect to the (arbitrary) upper limits of integration, substituting the definition of $\hat{c}\left(s\right)$, and isolating the determinant gives a constraint on the relationship between the two distributions, and therefore a constraint on ***f***(***s***):

$$\begin{array}{c} p(s)/u(s)\;=\;|J(s+f(s))|\;=\;|I+J(f(s))|. \end{array}$$ (42)

where ***I*** is the identity matrix. Here we have substituted *u*(***s***) for $u(\hat{c}\left(s\right))$ because *u* is a constant function and the argument has no effect on its value. The limits of the multiple integration are a vector, and the derivative has been taken with respect to each element.

Note that Eq (42) is under constrained because each component of the vector $f\left(s\right)\in R^{k}$ is an unknown, whereas the left hand side is a scalar value. However, recall from the 1-D derivation that ***f***(***s***) is constrained to be a smooth, continuous function of ***s***. This implies that both the sensory and stimulus spaces, $\hat{s}$ and ***s***, are defined over continuous coordinate systems that are simply connected and do not have coordinate singularities—an assumption that has been implicit in prior work, where smooth 1-D distributions *p*(*s*) have been integrated into a cumulative density function to transform coordinates between the stimulus and sensory spaces. We will show that these mild constraints imply that ***f***(***s***) is a conservative field in higher dimensions, and can therefore be described as the gradient of a scalar function. This reduces the number of unknowns to one at each point ***s***. We refer to this scalar function as the encoding potential: the gradient of this function yields the mapping that is needed to acheive the optimality criteria derived in the preceding sections.

For ***f***(***s***) to be conservative, its integral along an arbitraty path ***r***(*t*) through stimulus space must not depend upon the path taken, but solely on the endpoints. Here, *t* denotes the variable parameterizing the path. To write the line integral along the path, note that $f\left(s\right)=\hat{c}\left(s\right)-s$ is the difference between two coordinates. Let ***c***(***s***) define the stimulus coordinates similarly to the sensory coordinates. That is, ***s*** = ***c***(***s***), where ***c***(***s***) is simply the identity function. Thus, $f\left(s\right)=\hat{c}\left(s\right)-c\left(s\right)$. This additional notation is adopted to make the meaning of the line integral clear:

$$\begin{array}{c} \int f\left(r\left(t\right)\right)\cdot r^{\prime }\left(t\right)dt\;=\int [\hat{c}\left(r\left(t\right)\right)-c\left(r\left(t\right)\right)]\cdot r^{\prime }\left(t\right)dt. \end{array}$$ (43)

Here, the dot is used to denote the dot product. For the line integral to be independent of the path, ***r***(*t*), it must be integrable. The second coordinate function, ***c***(***s***), integrates trivially. Let *γ*(***s***) refer to this stimulus coordinate potential:

$$\begin{array}{c} \int c\left(r\left(t\right)\right)\cdot r^{\prime }\left(t\right)dt\;=\frac{1}{2}s^{2}+C\;\equiv \;\gamma \left(s\right). \end{array}$$ (44)

where $r^{\prime }\left(t\right)=\frac{dr}{dt}$ and *C* is a constant of integration. Whether ***f***(***s***) is conservative therefore depends upon the integrability of the sensory coordinate function, $\hat{c}\left(s\right)$.

We can ensure $\hat{c}\left(s\right)$ to be Riemann integrable by leveraging a constraint that has been implicit in prior work [9]: the sensory coordinate function is constrained to be continuous and thus integrable. Note that this introduces a constraint on the coordinates, rather than the functions defined on them. Let $\hat{\gamma }\left(s\right)$ refer to this sensory coordinate potential:

$$\begin{array}{c} \int \hat{c}\left(r\left(t\right)\right)\cdot r^{\prime }\left(t\right)dt\;\equiv \;\hat{\gamma }\left(s\right). \end{array}$$ (45)

The line integral of the displacement field ***f***(***s***) can therefore be described by a scalar potential *v*(***s***):

$$\int f\left(r\left(t\right)\right)\cdot r^{\prime }\left(t\right)dt=\hat{\gamma }\left(s\right)-\gamma \left(s\right)\;\equiv \;v\left(s\right)$$ (46)

f(s)=∇v(s). (47)

Here, ∇*v*(***s***) denotes the gradient of the scalar potential. We can now rewrite Eq (42) with the substitution ***f***(***s***) = ∇*v*(***s***):

p(s)/u(s)=|I+J(∇v(s))| (48)

$$\begin{array}{cc} & =|I+H(v(s))|, \end{array}$$ (49)

where ***I*** is the identity matrix, and H(⋅) denotes the Hessian operator. Note that this equation now contains only one unknown (the scalar value of *v*(***s***) at each point in space), and holds regardless of the dimensionality of the spaces.

In summary, constraining the transformed sensory coordinates, $\hat{s}$, to be continuous implies that the line integral in Eq (45) can be evaluated for all paths, which in turn implies that the underlying displacement field ***f***(***s***) can be written as the gradient of a scalar potential function—and conversely, constraining ***f***(***s***) to be the gradient of a scalar function implies that the coordinates $\hat{s}$ are continuous. In the next section we show that Eq (49) can be solved for the potential *v*(***s***), from which we recover ***f***(***s***). The optimal neuronal population density is therefore proportional to the density of the stimulus as predicted in Eq (38).

### Solving for the optimal mapping function in 2-D

The work laid out in the previous sections advances our ability to predict the properties of neuronal populations that efficiently encode more than one stimulus dimension. In practice, the characterization of neuronal tuning curves beyond 1-D requires exponentially increasing amounts of data collection in the laboratory. While large datasets of multidimensional tuning curves are becoming more feasible due to the development of high-throughput data collection paradigms, as a next step we will focus on making predictions for 2-D stimuli, because these are most likely to be relevant for experimental confirmation in the near term.

We will denote the separate dimensions of a 2-D stimulus ***s*** as *s*_1_ and *s*_2_. For 2-D stimuli, the determinant in Eq (49) expands to give the following differential equation:

$$\begin{array}{c} p\left(s\right)/u\left(s\right)=\frac{\partial^{2}v}{\partial {s_{1}}^{2}}\frac{\partial^{2}v}{\partial {s_{2}}^{2}}-{\left(\frac{\partial^{2}v}{\partial s_{1}\partial s_{2}}\right)}^{2}+\frac{\partial^{2}v}{\partial {s_{1}}^{2}}+\frac{\partial^{2}v}{\partial {s_{2}}^{2}}+1. \end{array}$$ (50)

A numerical method for solving this equation is given in [27]. Briefly, the optimization procedure initializes *v*(***s***) to the zero matrix (no transformation) at a fixed resolution. This initial estimate is then refined to minimize the total squared difference between the right and left hand sides of Eq (50), accumulated across the discretized domain (See [27], Equations (17)–(23).) Numerical refinement proceeds using the non-linear conjugate gradient method of [37] and computations are made efficient using optimal, separable filters derived by [38]. The optimization is wrapped in a multi-grid scheme in which a low resolution potential is first estimated.

The discretized domain is defined within a finite square boundary. Geometric properties of the potential are used to constrain the numerical solution within this boundary. Specifically, the potential must obey boundary constraints, or probability mass will be mapped outside of its finite domain (mass must be conserved under ***f***(***s***)). Components of ∇*v* that are orthogonal to the boundary must be zero at the boundary. The potential must therefore become flat as one approaches the boundary, but can be sloped as one travels along the boundary. This constraint is enforced by repeating the values of *v*(***s***) within a fixed perpendicular distance of the boundary. The fixed distance is the width of the discrete convolutional operators, guaranteeing that the perpendicular component of the discrete gradient is zero at the boundary. In the optimization, these repeated values are changed as a group to enforce this constraint.

Lastly, the above optimization as-is would force the domain boundary to be square. To allow for different boundary shapes, the uniform distribution can be defined in a smaller interior region, such as a circle. Outside of this region, both probability distributions are defined to be equal to avoid introducing discontinuities. After optimization, the gradient field is cropped to the boundary of this region. An implementation of this optimization in Matlab code is publicly available (https://github.com/eacooper/MultidimensionalEfficientCoding). Note that the numerical optimization does not guarantee that the solution is unique. Multiple minima have not, however, been observed while testing with a variety of probability distributions. Once *v*(***s***) is found, its gradient gives the displacement field ***f***(***s***).

### Summary of constraints and assumptions

Thus far, a variety of constraints, assumptions, and approximations have been chosen. Here, we will summarize and discuss some of the key choices and the limitations they introduce for predicting optimal heterogenenous neuronal populations. In the following sections, we will then apply this framework to example 2-D stimulus distributions and discuss some common features of the optimized encoding populations.

First, we make a number of choices about the initial uniform neuronal population in stimulus space. The neuronal tuning curves are assumed to be unimodal so that they can tile the stimulus space and the approximation in Eq (8) can hold. This assumption is incompatible with monotonically increasing tuning curves. For example, the population-level activity for uniformly spaced sigmoidal tuning curves increases in the direction that the sigmoids are increasing, resulting in quite different populations that satisfy the constraint on expected firing rate, as discussed in [9]. Even restricting ourselves to unimodal tuning curves, the final appearance of the optimal heterogeneous encoding population is affected by the exact shapes of these tuning curves, as well as the original density with which they tile the space. This issue is also true in prior 1-D work, in which one must manually select various features of the uniform population (the number of neurons, their initial tuning curve shapes, and their amount of overlap) in order to visualize an optimized population [9]. However, the extension into multiple dimensions necessitates additional choice-making. For example, in addition to the population density, one must also choose the lattice over which the uniform population is defined. We chose a hexagonal lattice in the simulations below, but similar results were obtained with a rectangular lattice. The initial lattice will affect the final positions of individual neurons in the optimized population—where they start affects where they end up. We assume that most biological neuronal populations are in a high density regime and should thus be evaluated at a population level, rather than based on the positioning of individual neurons. Another choice that becomes less constrained in higher dimensions is the shapes of the tuning curves. For example, in the following simulations we chose isotropic 2-D tuning curves, but non-isotropic shapes could satisfy Eq (8) equally well. The choice of the initial tuning bandwidth in each dimension is thus left unconstrained. Importantly, these choices will change the specific details of the predicted optimal population, but they will not result in populations that disobey the core predictions of the theory: an optimal population places proportionally more neurons with narrower tuning in regions of the stimulus space that are more likely to occur and does not modify their gain.

Second, only two functions are adopted to parameterize the heterogeneous population in terms of a transformed version of this uniform population. These functions are the displacement field and the gain function. The simplicity of this parameterization limits the ultimate shape of the heterogeneous population, and also requires constraints on these functions in order to reach a closed form solution. Specifically, these functions are constrained to be continuous and slowly changing with respect to the tuning curves. These constraints ensure that Eqs (16) and (34) are valid approximations of the heterogeneous population’s Fisher information, and the solution will thus hold more exactly when tuning curves are relatively narrow. These constraints are carried over from the 1-D framework that we build on, but the constraint that the displacement field be continuous takes on new significance in higher dimensions [9]. In 1-D, this continuity constraint on displacement was implicit because the *warping function* was defined by the cumulative, an integral of a density function that was itself continuous [9]. In the multidimensional case, the continuity constraint implies that the optimal displacement field must be conservative. As described in the previous section, the fact that the displacement field is conservative means that it can be written as the gradient of a scalar function and is curl free (this is the outcome of Eqs (43)–(47)). This conservative property allows us to obtain a numerical solution, but it limits our approach to modeling neuronal populations that represent continuously valued stimulus variables. For the current formulation, this constraint provides a useful starting point and importantly enables us to derive detailed predictions for populations that encode multiple stimulus dimensions. But developing models that move away from the closed-form framework used here may provide new insights into the variety of multidimensional schemes that can lead to efficient neural representations.

Lastly, the numerical method for solving for the potential function (whose gradient defines an optimal displacement field) introduces additional assumptions. Eq (50) defines the stimulus distribution and the sensory distribution (the latter being uniform) over the same domain. Because the two domains are related by the addition of a displacement field, they are required to share the same units and metric, and the left hand side of the equation is only well defined if the two domains overlap. However, the choice of domain does not change the optimal solution arbitrarily. For example, we can consider a change of units or scale in one stimulus dimension. If the range is initially 0-1 kHz but becomes 0-1000 Hz, this will change both the range of the domain in one dimension and also will generally change the geometry of the 2-D domain. Importantly, the solution to Eq (50) is invariant under such a domain transformation, after applying the same change of units to the potential function, and thus does not vary arbitrarily with a change to the geometry of the domain. In contrast, the shape of the domain does come into play when the numeric optimization is implemented. The main observed effect of the boundary shape is to change the tuning curve shapes near the boundary. We use a simple circular or square boundary in all simulations that follow. Similar issues arise in the 1-D cumulative distribution solution, in which tuning curves become constant in areas of the domain where the stimulus probability falls to zero. From a practical standpoint, because the choice of boundary is unconstrained and primarily affects the results near the boundary, we restricted our analysis to a smaller central square region of the stimulus space.

### Stimulus distributions and optimal neuronal populations in 2-D

**Fig 3:**
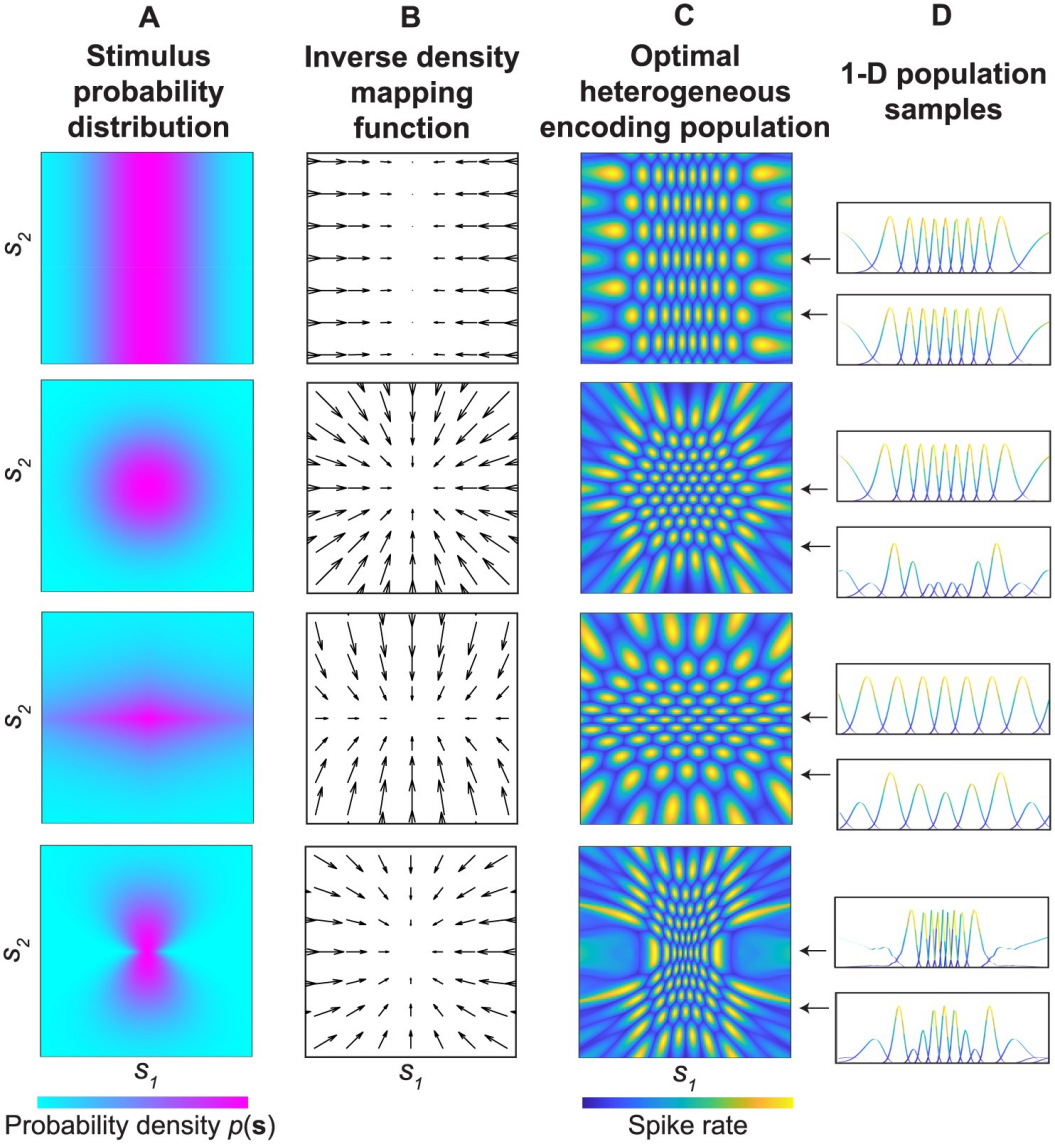
Example 2-D stimulus probability distributions and the resulting optimal encoding populations. A) Each row represents a different example probability distribution over two stimulus dimensions (*s*_1_ and *s*_2_). For each panel, the probabilities are defined over a lattice ranging from -1 to 1 (cropped to the central 65% to remove boundary artifacts). Top: uniform over *s*_2_ and Gaussian distributed over *s*_1_ (*μ* = 0, *σ* = 0.25). Upper middle: isotropic bivariate Gaussian (*μ* = 0, *σ* = 0.25 in both dimensions). Lower middle: bivariate generalized Gaussian (*μ* = 0, *σ*_*s*1_ = 0.75, *σ*_*s*2_ = 0.25, *power* = 1.1). Bottom: Gaussian distributed over *s*_1_, with a *σ* that varies non-linearly with *s*_2_ (this distribution is non-separable). B) For each probability distribution, we show a down-sampled and scaled visualization of the inverse density mapping function. The direction and length of the arrows illustrate how density will be mapped from sensory space into the stimulus space. C) For each probability distribution, we show an example neuronal population that has been warped to optimally encode the stimulus. For these visualizations, we chose a population of neurons with isotropic bivariate Gaussian tuning curves (*σ* = 0.05) tiling the space on a hexagonal lattice (*spacing* ≈ 0.2). Though these choices for the population are arbitrary, varying them does not change the qualitative properties of the warped populations. Circular domain boundaries were used for the bottom three examples. To account for the uniform probability in panel A (top row), the population illustrated in panel C (top row) was defined with a square rather than a circular domain boundary. D) On the right side of each population, a pair of 1-D samples are illustrated. For each sample, *s*_2_ is held constant and the tuning curves are visualized over *s*_1_. Neurons with a maximum normalized response of less than 0.2 within the sample are not visualized. The distribution of Fisher information in each 2-D population is shown in S2 Fig).

Given any 2-D stimulus probability distribution, the method described in the previous section allows us to find a displacement field with which to warp a neuronal population in order to efficiently encode the stimulus (i.e., with neuronal density proportionate to probability). In this section, we explore the properties of neuronal populations optimized with this method (Fig 3). Fig 3A illustrates four 2-D stimulus probability distributions. One distribution is Gaussian over *s*_1_ and uniform over *s*_2_ (top), one distribution is an isotropic bivariate Gaussian (upper middle), one distribution is an anisotropic leptokurtotic generalized Gaussian (lower middle) and one distribution is Gaussian over *s*_1_ with a standard deviation that varies non-linearly with *s*_2_ (bottom). For each 2-D stimulus probability distribution, we also show the inverse of the numerically optimized displacement field, which intuitively illustrates the warping of neuronal tuning curves (Fig 3B). Generating visualizations of the warped populations involves some choices, the most obvious of which is the shape of the unwarped 2-D tuning curves. For these examples, we chose bivariate isotropic Gaussians for the unwarped tuning curves, though any shape that can reasonably tile the stimulus space is possible. The resulting warped 2-D populations are shown in Fig 3C. Finally, for each population, two example 1-D samples are shown (Fig 3D). These samples show what the 1-D tuning curves in this population look like as a function of *s*_1_ assuming *s*_2_ was held constant at two different values. As described in the previous section, only the central region of the stimulus domain is analyzed in each panel due to limitations of the numerical method at the boundary.

Several features of the optimized populations are qualitatively notable. First, the density with which tuning curves tile a particular region of the space is proportional to the probability density in that region, as required by Eq (38) [8, 9]. Because the population is constrained to maintain a fixed overlap between neurons, the widths of the tuning curves in the population are thus heterogeneous. Qualitatively, the bandwidth appears narrower (sharper tuning curves) in areas of high probability, and broader in areas of low probability. However, the bandwidth can also vary substantially for *s*_1_ and *s*_2_. With respect to the maximum firing rates of the neurons, following from the fact that the optimal gain function is uniform, each neuron has the same peak firing rate associated with its 2-D tuning curve (Eq (39)). S2 Fig illustrates the measured distribution of Fisher information in each of the example populations in Fig 3C, and shows that these distributions follow the square root of the stimulus probability density as expected by Eq (34). Thus, if a population is optimal in the sense described here, it would be possible to predict the natural distribution of the stimuli being encoded from the Fisher information measured from the population responses. Such an inverse efficient coding scheme is similar to a method proposed in [39], which derives the stimulus distribution from the distribution of firing thresholds in an optimal population of spiking neurons.

**Fig 4:**
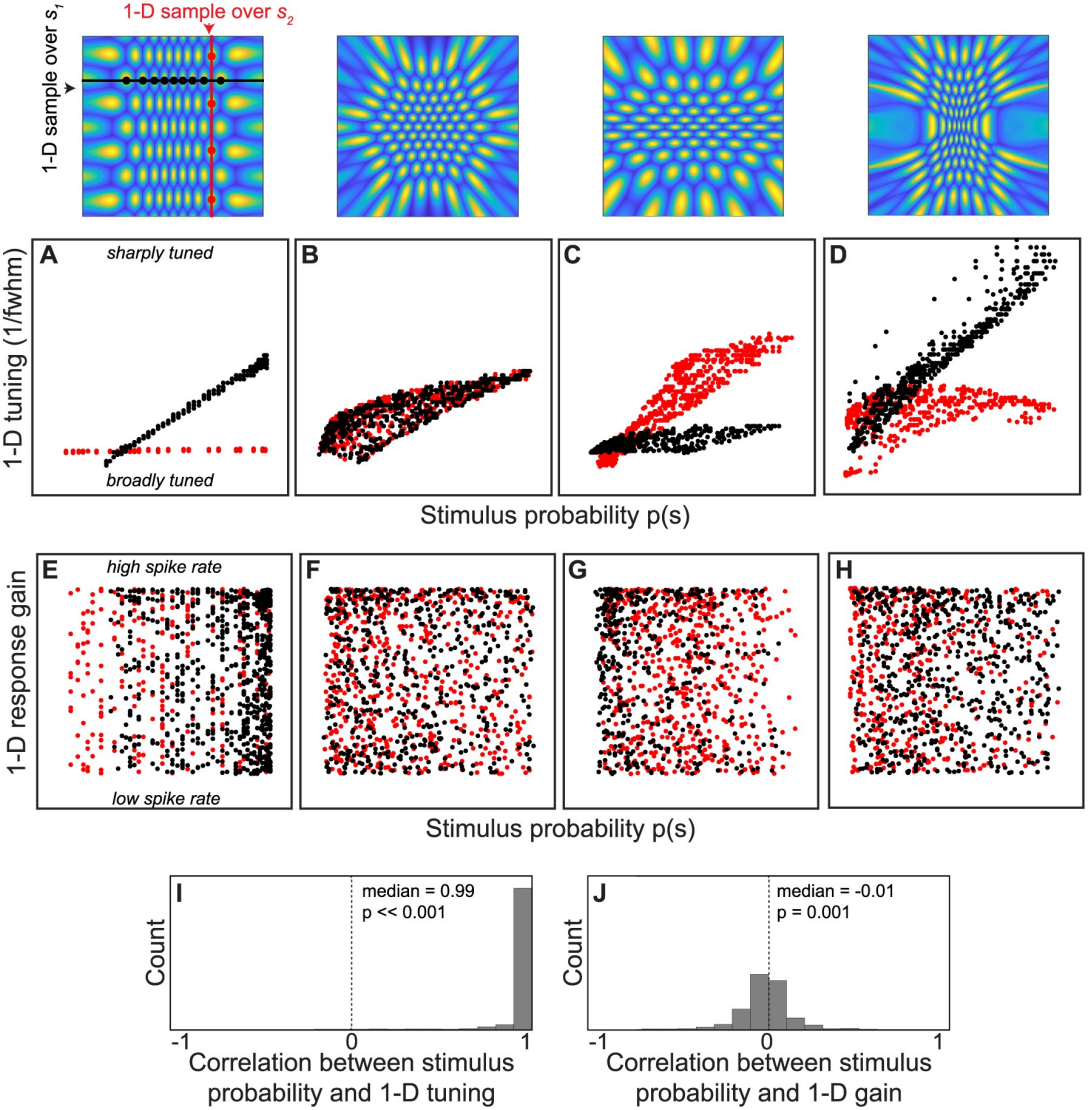
Analysis of how lower-dimensional measurements of tuning curve properties (1-D gain and tuning width) relate to the higher-dimensional stimulus probability. Four example neuronal populations are shown, which correspond to the probability distributions and optimized mappings in Fig 3 (re-plotted in the top row). We simulated a set of 1-D experiments by selecting a single value for either *s*_1_ or *s*_2_ and measuring the response gain (maximum response) and tuning sharpness (inverse of the full width half maximum) of a set of neurons within this ‘slice’ (*σ* pre-warping was 0.05). This method simulates what the measured neuronal gain and tuning bandwidth would be in an experiment in which one stimulus feature was held constant and the other was varied. (A-D) For each of the illustrated populations, these panels plot the 1-D tuning sharpness as a function of probability, for a sample of neurons (400-700 neurons). Samples that were drawn by holding *s*_1_ constant are shown in red, and samples drawn by holding *s*_2_ constant are in black. (E-H) These panels plot the 1-D response gain as a function of stimulus probability, as in the panels above. (I,J) We repeated these simulations 500 times for randomly generated 2-D stimulus probability distributions and calculated the correlation between gain/tuning and probability. Each probability distribution was a zero-centered, bivariate Gaussian with a random orientation and major/minor *σ* drawn uniformly from 0.1-0.4. For each simulation, the tuning curves were modeled as isotropic Gaussians with *σ* drawn uniformly from 0.03-0.07. A random 1-D slice was selected, and 25 neurons were sampled. P-values indicate the results of a Wilcoxon signed rank test determining whether the median correlation was significantly different from zero.

Because empirical measurements of neurons often characterize a subset of their tuning properties, we next conducted simulations designed to examine how measurements of lower dimensional (1-D) tuning properties relate to the higher-dimensional (2-D) stimulus probability distribution encoded by example populations (Fig 4). These simulations can reveal insights into how to evaluate efficiency when the full dimensionality of the neuronal tuning curves (and the encoded sensory space) is unknown. Specifically, starting with each of the example neuronal populations illustrated in Fig 3C, we took a set of 1-D samples through each population, determining the 1-D tuning curves that would be obtained by holding either *s*_1_ or *s*_2_ constant and measuring neuronal spike rate as a function of the other variable. We then summarized each of these 1-D tuning curves by their overall gain (maximum response) and tuning sharpness (inverse of full width at half maximum response). In practice, experimenters may try to identify the tuning preference for each neuron along one dimension before characterizing the tuning for another (for example, identifying the preferred stimulus orientation of a visual neuron before characterizing its tuning curve for spatial frequency).

First, we asked whether measurements of 1-D tuning sharpness bear a consistent relationship to 2-D stimulus probability. Based on data from several neurophysiological studies, previous work found consistent evidence that neurons encoding more likely stimuli are more sharply tuned, however, only 1-D stimulus probabilities were considered [15]. Does the encoding of a higher dimensional stimulus space affect this prediction? In Fig 4A–4D we plot the 1-D tuning for a sample of neurons from each example population in Fig 3, as a function of the stimulus probability (*p*(**s**), which is the joint probability of *s*_1_ and *s*_2_). While there is some variability, we can see that 1-D tuning generally follows a trend of having broadly tuned neurons encode low probability values, and sharply tuned neurons encode high probability values. The one exception is when *s*_2_ is uniformly distributed and *s*_1_ is not (Fig 4A, red circles). In this case, neurons are predicted to maintain a relatively constant bandwidth of tuning in *s*_2_ (reflecting the uniform distribution) even when the joint probability of *s*_1_ and *s*_2_ is variable. However in practice it may be rare to encounter natural stimulus features that truly follow a uniform probability distribution.

In a follow up analysis, we generated 500 random stimulus probability distributions by randomly varying the aspect ratio and orientation of a bivariate Gaussian distribution. We then sampled 25 neurons from an optimal encoding population for each random distribution and calculated the tuning sharpness along a single stimulus dimension. We calculated the correlation between the stimulus probability density (*p*(**s**)) and the tuning sharpness associated with each neuron. Fig 4I shows a histogram of these correlations, which tended to be greater than zero, and typically close to one (median = 0.99). A Wilcoxon signed rank test indicated that the median was significantly greater than 0 (p ≪ 0.001). The results of this simulation suggest that the correlation between tuning sharpness and probability is a general property across a range of stimulus distributions. Repeated runs of this simulation always produced similar results.

A separate insight arises when considering the prediction that efficient neuronal encoding populations have uniform gain. Previous work has examined the uniform gain prediction in 1-D by examining the maximum spike rate of neurons sampled from several different brain regions [15]. The authors found substantial variability of gain within populations that nominally encode the same stimulus feature, seeming to violate the uniform gain prediction. This discrepancy might suggest that gain is affected by other factors that do not relate to coding efficiency. It has also been pointed out that applying alternative tiling constraints yields a prediction that gain and probability should be positively correlated [30]. We wondered whether the observed variability in response gain might be expected from our simulation. For example, the right-most column in Fig 3 shows several 1-D samples through each 2-D population. If neuronal preferences for stimulus feature *s*_1_ are measured empirically by varying *s*_1_ and holding stimulus feature *s*_2_ constant (as is often done in neurophysiological experiments), the resulting 1-D tuning curves would appear as illustrated in the insets. It is clear from these insets that the resulting data can appear as if neuronal gain is variable within this population, even though we know it is not. This apparent variability occurs simply because some neurons’ peak responses to *s*_1_ lie along different values of *s*_2_, and vice versa. In Fig 4E–4H, we plot the 1-D response gain obtained by taking a set of 1-D samples through each example population. Similar to the empirical physiological results, we see that the predicted response gain varies between neurons, and this variability bears no consistent relationship to probability [15].

In a follow up analysis, we again varied the aspect ratio and orientation of a bivariate Gaussian probability distribution and now measured the linear correlation between probability and gain. The results suggest no consistent relationship between 1-D response gain and stimulus probability encoded by individual neurons (Fig 4J). The median correlation value was -0.01. While this median was significantly different from zero as determined by a Wilcoxon signed rank test (p = 0.001), correlation values above 0.5 were highly unlikely. This observation suggests that in order to test predictions for any systematic patterns of gain (e.g., uniformity, correlation with probability), a more exhaustive knowledge of the stimulus features being encoded may be necessary, even if those features are statistically independent. This knowledge may be prohibitive in practice for neuronal populations that robustly encode a large number of stimulus dimensions, but could be used as a guideline for lower-level populations. At the least, future empirical work can determine gain by varying several features of a stimulus and characterizing the a multidimensional tuning curve.

Viewing 2-D populations also allows for new observations and predictions about efficient populations in higher dimensions. In addition to heterogeneity of 1-D tuning width, we see that the predicted efficient populations vary substantially in bandwidth around their peak in *s*_1_ and *s*_2_. For example, in each population, there are some neurons that are sharply tuned for *s*_1_, but weakly tuned for *s*_2_ (very broad tuning curves), and vice versa. Thus, we predict that optimal populations may contain a combination of neurons jointly tuned for each dimension, in addition to some tuned just for one dimension.

Finally, we observed that neurons in these optimal populations can exhibit heterogeneity of separability. That is to say, in many cases the efficient population contains some tuning curves that are largely separable in their tuning for *s*_1_ and *s*_2_ (i.e., the preferred value of one variable does not change much as the other is varied) and others that are not. Additionally, non-separable tuning curves may arise in the efficient population regardless of whether the input distribution is itself separable. For example, the bivariate Gaussian stimulus distribution in Fig 3 (upper middle row) is separable in *s*_1_ and *s*_2_, but produces a number of non-separable neurons (i.e., their tuning over *s*_1_ varies as a function of *s*_2_). The presence of both separable and non-separable curves in the efficient population is interesting when considering the problem of decoding the population responses. For sub-populations of neurons that have separable tuning curves, a single variable value can be decoded from their response without considering the value of the other stimulus dimension(s). This is because variations in the irrelevant stimuli only modulate the magnitude of each neuron’s response [40]. Furthermore, if we make the observation that for many of the probability distributions we have tested the non-separable tuning curves tend to appear predominantly in regions of lower probability, and if we assume the decoding population employs a simple independent read-out method, in this scheme it appears that not only are low probability regions of the stimulus space coded with less precision (tuning curves are broader), but they also may be decoded less accurately.

## Discussion

Notable theoretical advances in neuroscience have come from applying the efficient coding framework to signals derived from the natural environment (e.g., natural images, videos and sounds), which tend to be non-uniformly distributed. In several cases, this approach produces predictions about the distribution of neural resources that have been confirmed with physiological measures (e.g., [14, 15, 36, 41, 42]). Early theoretical work considered how single neurons or uniform/homogeneous populations encode sensory variables [33, 36, 43, 44]. Some of these original models explicitly considered the case of multidimensional stimuli [26]. More recent studies, motivated by the observation of substantial variability in the tuning functions of neurons in the same population, have investigated how heterogeneous populations of tuning curves can efficiently encode 1-D stimuli [8, 9, 12, 30]. However the added complexity of multidimensional stimuli has made it difficult to generalize these heterogeneous population models to higher dimensions.

Here we have addressed this gap by providing a theoretical prediction for how neural resources should be allocated in the context of a heterogeneous population that is used to simultaneously encode an arbitrary number of stimuli. In particular, we present a closed-form solution for the optimal neuronal tiling density. This solution suggests that regions in stimulus space that are more likely should be represented by a proportionally larger number of neurons, which are each proportionally more selective. This result is a natural extension of previous work on the neural coding problem, which considered heterogeneous populations of tuning curves that respond to 1-D stimuli [8, 9]. Our results also suggest that each neuron should exhibit the same maximum response. The key intuition behind this result is that each neuron in the optimal population is equally likely to fire a spike under natural conditions (that is, when the presented stimuli come from the environmental probability distribution).

The import of any theoretical model often hinges on its ability to be verified experimentally. The prediction that neural resources simultaneously encoding multiple dimensions should be distributed proportionally to the joint environmental distribution could be experimentally verified or falsified with a combination of empirical measurements of multidimensional tuning curves and environmental statistics. Interestingly, considering the case of multidimensional stimuli revealed that the prediction that the response gain should be constant throughout such a population will likely be difficult to test experimentally. Testing that prediction would require exhaustive knowledge of all features being coded by the population, in order to rule out the possibility that observed non-uniform gain may be the result of warping along an uncontrolled stimulus dimension. Thus, we have shown that predictions made by efficient coding models can be deceptively simple, and that it is important to consider how unmodeled features of biological function (i.e., multidimensional tuning) may manifest themselves during experimental observation. In addition to these explicit predictions, this work makes it possible to ask questions about the computational benefit of utilizing an optimal multidimensional population as opposed to multiple one dimensional populations when performing downstream tasks such as decoding. For example, in [9] the density of the encoding population implicitly encodes a prior distribution during the decoding process. So the use of two 1-D populations instead of a 2-D population would encode the best separable approximation of the true joint probability distribution, which would presumably be suboptimal. The extent to which separable approximations affect decoding performance would be an interesting avenue for future research.

Like all optimization frameworks, ours makes several assumptions, fixing certain aspects of the model based on some features of biological networks and allowing others to vary during the optimization step. For example, in the current framework, the optimal mapping between input stimulus space and the sensory representation is independent of the initial pattern of tuning curves, so long as they meet the assumptions laid out in the model. It is important to note that related population coding formulations (whether they choose different features to vary or adopt different constraints) can make quite different predictions about the allocation of neural resources. This highlights that there are multiple paths to achieving coding efficiency even in the framework presented here [12, 30]. Other approaches such as sparse coding and independent components analysis address a similar class of questions about optimality in higher dimensions. Critically, our extension to multidimensional heterogenous populations allows for more direct comparisons with these and other coding schemes for higher dimensional signals. The availability of more datasets characterizing neuronal responses across multiple stimulus dimensions should eventually answer the questions of what real neuronal systems are optimized for and what constraints they operate under.

## Supporting information

S1 Fig Importance of assumptions about displacement field and gain function when parameterizing heterogeneous neuronal populations. The parameterization is illustrated as in Fig 2, however the displacement field and gain functions (C,E) now vary substantially within the bandwidth of individual neuronal tuning curves. When this is the case, the approximation of Fisher information is no longer accurate (G).
(TIF)

S2 Fig Recovering stimulus distribution from efficient populations. For each example probability distribution from Fig 3 (A) and numerically optimized population (B), the measured Fisher information associated with the population is plotted as the determinant of the Fisher information matrix (C,D) (Eq (29)). Each panel is scaled to the maximum, which is indicated in the bottom right. The Fisher information pattern reflects the shapes and distribution of the tuning functions, which here are warped from a population of bivariate Gaussians on a hexagonal sampling lattice (*σ* = 0.05). In (C), neurons were relatively sparsely spaced as illustrated in (B), resulting in irregularities in the measured Fisher information (*spacing* ≈ 0.20). In (D), this spacing was decreased by a factor of 2 to illustrate the smooth Fisher information. Because the determinant of the Fisher information matrix is proportionate to the squared probability of the stimulus, the results in (D) can be used to estimate the stimulus probability from the neuronal population directly (E). The panels illustrate that the numeric optimization results in a population in which the 2-D Fisher information is allocated appropriately for the input stimulus probability. In all panels, the stimulus space is cropped to +/- 0.65 to remove boundary artifacts resulting from the numeric optimization.
(TIF)
