# Supplementary figures and images for: Efficient sensory coding of multidimensional stimuli
Source: PLoS Comput Biol. 2020 Sep 24;16(9):e1008146. doi: 10.1371/journal.pcbi.1008146 (PMC7514067; doi:10.1371/journal.pcbi.1008146)

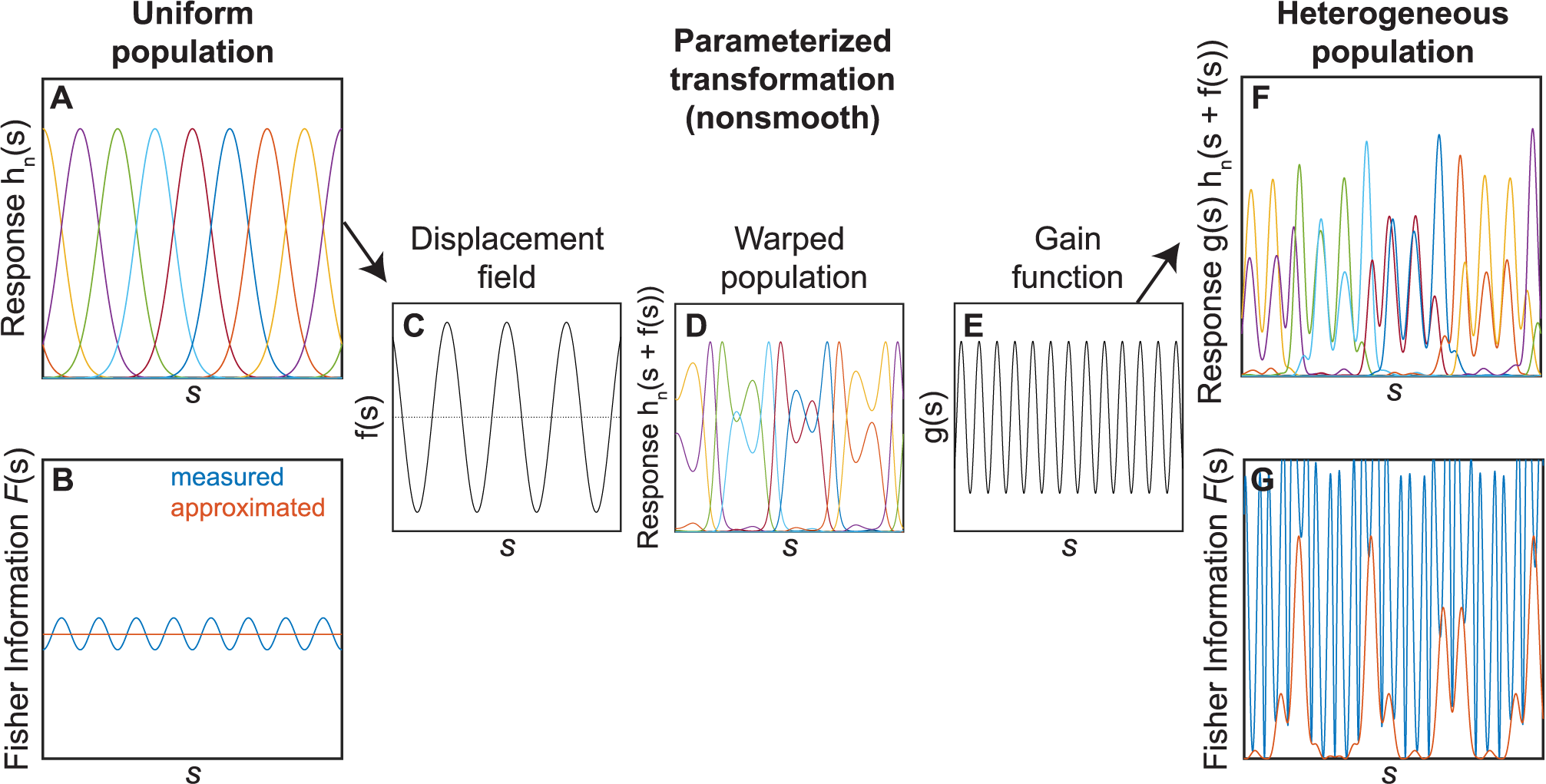

Supplement: S1 Fig — The parameterization is illustrated as in Fig 2, however the displacement field and gain functions (C,E) now vary substantially within the bandwidth of individual neuronal tuning curves. When this is the case, the approximation of Fisher information is no longer accurate (G). (TIF) [file pcbi.1008146.s001.tif]

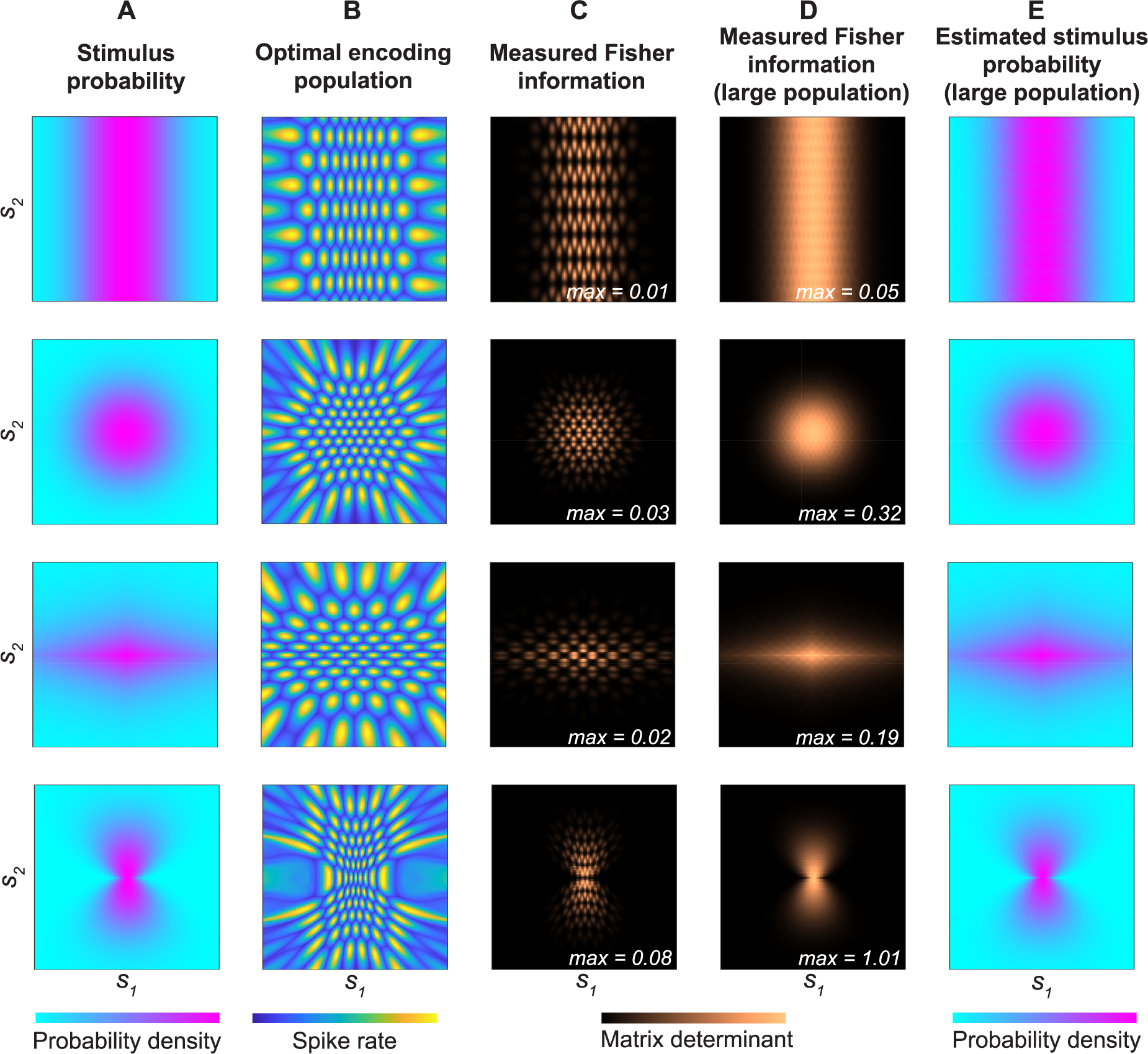

Supplement: S2 Fig — For each example probability distribution from Fig 3 (A) and numerically optimized population (B), the measured Fisher information associated with the population is plotted as the determinant of the Fisher information matrix (C,D) (Eq (29)). Each panel is scaled to the maximum, which is indicated in the bottom right. The Fisher information pattern reflects the shapes and distribution of the tuning functions, which here are warped from a population of bivariate Gaussians on a hexagonal sampling lattice (σ = 0.05). In (C), neurons were relatively sparsely spaced as illustrated in (B), resulting in irregularities in the measured Fisher information (spacing ≈ 0.20). In (D), this spacing was decreased by a factor of 2 to illustrate the smooth Fisher information. Because the determinant of the Fisher information matrix is proportionate to the squared probability of the stimulus, the results in (D) can be used to estimate the stimulus probability from the neuronal population directly (E). The panels illustrate that the numeric optimization results in a population in which the 2-D Fisher information is allocated appropriately for the input stimulus probability. In all panels, the stimulus space is cropped to +/- 0.65 to remove boundary artifacts resulting from the numeric optimization. (TIF) [file pcbi.1008146.s002.tif]
